# Epidemiology and Outcomes of Intramedullary Nailing for Tibial Diaphyseal Fractures: A Retrospective Multicenter Cohort Study

**DOI:** 10.7759/cureus.82894

**Published:** 2025-04-24

**Authors:** Fábio Lucas Rodrigues, Ana Lya Moya Ferrari, Fernando Ferraz Faria, Rafael Luiz Emmanoel Pinto, Manuela Fernandes Lopes, Maria Eduarda Alencar Santos, Evelyn Cardenas Varela, Manuel Jucelino Lopes Filho, Marianna Nogueira Cecyn, Nelson Henrique Carvalho De Oliveira

**Affiliations:** 1 Orthopedics and Traumatology, Santo André, São Bernardo do Campo, and São Caetano do Sul (ABC) Medical School, Santo André, BRA; 2 Research and Development, Biomecanica, Jaú, BRA; 3 Orthopedics and Traumatology, Cajuru University Hospital, Curitiba, BRA; 4 Orthopedics and Traumatology, Adriano Jorge Hospital Foundation, Manaus, BRA; 5 Psychobiology, Federal University of São Paulo, São Paulo, BRA

**Keywords:** consolidation, intramedullary fracture fixation, nail, shaft fractures, tibial fractures

## Abstract

Background: Intramedullary nails (IMN) are frequently used to fix tibial diaphyseal fractures. Despite good outcomes, complications such as infections, malunion, and nonunion may still occur, especially in low- and middle-income countries where healthcare resources and trauma care systems are often limited. This retrospective multicenter cohort study aimed to evaluate the epidemiology and clinical outcomes of IMN in tibial diaphyseal fractures in Brazil in terms of consolidation and complications.

Materials and methods: We analyzed data from 125 patients who underwent IMN for fixation of tibial diaphyseal fractures between August 2020 and January 2024 at two Brazilian hospitals.

Results: Traffic accidents were the most common injury mechanism (84%), and closed fractures accounted for 80.8% of the cases. Most patients (98.4%) achieved fracture consolidation within six months post-surgery, with a low complication rate (3.2%), including two nonunions with screw breakage, one malunion, and one infection. There was a significant association between previous external fixation and the type of fracture (closed and open). However, there was no significant association between previous external fixation and consolidation one year post-surgery.

Conclusion: The findings suggest that IMN is effective in achieving consolidation and low complication rates in Brazilian hospitals.

## Introduction

Lower limb fractures, including those of the patella, tibia, fibula, and ankle, are the most common bone injuries [1], with tibial diaphysis fractures presenting an incidence of 16.9 per 100,000 individuals per year [2]. The epidemiology of tibial fractures varies among the studied populations. While studies from Europe and North America reported a higher incidence of fractures caused by simple falls [3], studies from low- and middle-income countries reported traffic accidents as the primary trauma mechanism [4,5]. In those areas, the challenges associated with tibial fracture treatment include limited trauma care systems, scarce resources, insufficient healthcare professionals, and insufficient training and/or education of those healthcare professionals [6].

Intramedullary nails (IMN) are frequently used to fix tibial diaphyseal fractures, demonstrating good clinical outcomes with consolidation rates of 81% three months post-surgery and complete bone healing between six and nine months [7]. As a minimally invasive procedure, IMN preserves soft tissue and extraosseous blood supply [8]. Compared to external fixation, it promotes faster fracture union and recovery, while also reducing soft tissue complications and superficial infections [7,9,10]. IMN also results in fewer surgical site complications, including superficial and deep infections, and slightly faster healing when compared to open reduction internal fixation and minimally invasive percutaneous osteosynthesis [8,11].

In developed countries, IMN has replaced traditional fracture fixation methods, such as casting and external fixation, due to early mobilization and reduced complications from prolonged bed rest [6]. In Brazil, IMN is the prevalent treatment for tibial diaphyseal fractures [12]. However, complications such as delayed union (22.4%), nonunion (9.7%), and infection (8.1%) remain concerns among patients who underwent these procedures [9].

Although IMN is the gold standard treatment, its outcomes may vary depending on the characteristics of the population being studied. According to Pontin et al. [12], there is a lack of recent studies on the epidemiology of tibial diaphyseal fractures in developing countries, particularly Brazil. Previous Brazilian studies have compared IMN with bridge plates [13] and biplanar external fixation [14], investigating specific complications such as nonunion [5,15] or infection [16], or addressing fracture epidemiology without focusing on the effects of IMN [12,17].

Given the local context, the study's findings may better reflect patterns observed in other middle-income countries. Thus, our study aims to evaluate consolidation, overall complications, and epidemiological characteristics of tibial diaphyseal fractures treated with IMN in the Brazilian population.

## Materials and methods

The study followed the guidelines of the Brazilian National Council for Research Ethics. It was approved by the Ethics Committee of the ABC University Medical School (protocol number CAAE 82645524.7.1001.0082) on November 8, 2024, with written informed consent deemed unnecessary. This multicenter cohort analyzed retrospective data from tibial diaphysis fractures treated between August 2020 and January 2024 at two Brazilian institutions: Adriano Jorge Hospital Foundation, a public hospital in Manaus, AM, and Cajuru University Hospital, a teaching hospital in Curitiba, PR. Fractures were treated with either the Intramedullary Nail Orion SP Tibial or Intramedullary Nail SP2 Tibial (Biomecanica, Jaú, SP, Brazil). These institutions were recruited due to the history of use of this material. All patients underwent a reamed transpatellar tendon approach and received static locking IMNs. Rehabilitation was standardized, with progressive weight-bearing based on patient-reported pain levels.

The inclusion criteria were patients of both sexes, aged 18 years or older at the time of the surgical procedure, who underwent IMN for fixation of tibial diaphysis fractures, and with a minimum postoperative follow-up of one year. Patients aged 18 years or younger, those who did not complete the minimum follow-up, and patients with a previous history of fracture at the same bone were excluded.

The researcher responsible for the analysis created an online form using Google Forms (Google LLC, Mountain View, CA, USA) to collect data from patient medical records in a standardized manner. Four researchers from the Adriano Jorge Hospital Foundation and one researcher from Cajuru University Hospital were involved in data collection. Data was automatically tabulated into a spreadsheet and underwent a secondary evaluation by the researcher responsible for data analysis to ensure compliance with the inclusion and exclusion criteria. No information that could be used for patient identification was withdrawn from hospital systems. Only the researchers responsible for data collection and analysis had access to the table, which was stored anonymously to ensure confidentiality.

Demographic and clinical data included patient characteristics (gender, age, comorbidities, and history of substance use), fracture details (Müller-AO fracture classification, laterality, open or closed fracture, presence of multiple trauma, trauma in other systems, and mechanism of trauma), clinical history (initial treatment at another hospital, previous external fixation), outcomes (complications including infection, malunion, nonunion, or material failure), and fracture consolidation in six months and one year. An independent researcher, not involved in the surgery, assessed fracture consolidation by examining cortical integrity in at least three cortices on both anteroposterior and lateral radiographic views at six months and one year post-surgery.

For statistical analysis, categorical variables were summarized as absolute numbers (n) and relative frequencies (%), while numerical variables were described as means, standard deviations (SDs), and 95% confidence intervals (CIs). Chi-square and Fisher’s tests were used for categorical comparisons, with a 95% confidence level and a significance level of p<0.05. The analysis was performed using jamovi 2.4.11 (https://www.jamovi.org). Power analysis and real alpha were post hoc calculated for the exact Fischer test using G*Power version 3.1.9.7 (Heinrich-Heine-Universität Düsseldorf, Düsseldorf, Germany). Participants with insufficient follow-up information (less than one year) were excluded from the analysis.

## Results

Patient characteristics

**Figure 1:**
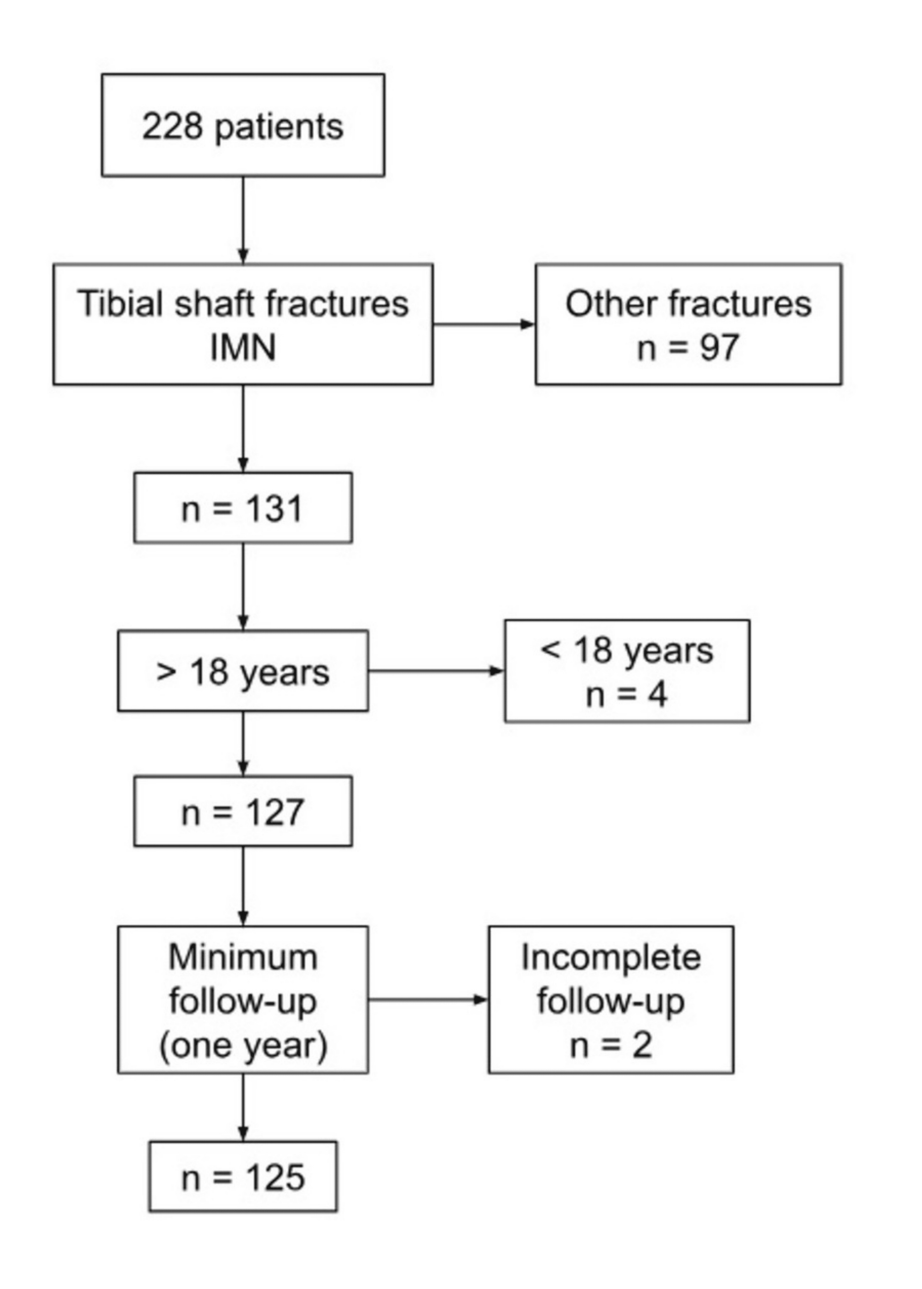
Patient inclusion and exclusion criteria IMN: intramedullary nail

During the data collection, 228 patients with lower limb fractures underwent IMN by trauma orthopedic teams at both institutions. Of these, 131 patients were treated for diaphyseal tibial fractures and satisfied the inclusion criteria. Four patients did not meet the minimum age and were excluded, leaving a sample of 127 patients. Two patients were excluded because they did not complete the minimum follow-up, resulting in a final sample of 125 patients (Figure 1).

The mean age was 33.37 ± 11.13 (Table 1). The sample consisted of 100 (80%) male patients. Most patients (n=121, 96.8%) had no comorbidities, while one (0.8%) had systemic arterial hypertension (SAH), and another one (0.8%) had both SAH and type II diabetes mellitus. Comorbidity data were unavailable for two patients (1.6%). A significant proportion of patients (n=101, 80.8%) reported using substances. Of those, 84 (83.17%) reported using alcohol and tobacco, and four (3.96%) were users of alcohol, tobacco, and illicit drugs. Only 11 (10.89%) patients consumed just alcohol, while two (1.89%) were illicit drug users only.

**Table 1:** Participant’s demographic data n = absolute frequencies, % = relative frequencies, SD: standard deviation

Sex	n (%)
Male	100 (80%)
Female	25 (20%)
Age	Mean (SD)
	33.36 ± 11.13
Substance use	n (%)
Yes	101 (80.80%)
No	24 (19.20%)
Type of substance (n=101)	n (%)
Only alcohol	11 (10.89%)
Only tobacco	0 (0.0%)
Only illicit drugs	2 (1.89%)
Alcohol and tobacco	84 (83.17%)
Alcohol, tobacco, and illicit drugs	4 (3.96%)

Injury characteristics

In our sample, 68 injuries (54.4%) occurred on the right side. Most fractures were classified as 42A (n=86, 68.8%), followed by 42B (n=35, 28%) (Table 2). Data analysis showed that most fractures (n=101, 80.8%) were closed, and most patients (n=102, 81.6%) had not undergone previous external fixation. A total of 98 patients (78.4%) initially received treatment at another hospital. There were no associated fractures in 106 cases (84.8%) and no injuries in other systems in 105 patients (84%). Thoracic injuries were reported in two patients (1.6%), and one patient (0.8%) had injuries in the abdominal system. Information about associated injuries in other systems was unavailable for 17 cases (13.6%). Traffic accidents were the most common trauma mechanism (n=105, 84%), exceeding all other causes combined. Sports trauma (n=4, 3.2%), physical assault (n=4, 3.2%), and ground-level falls (n=4, 3.2%) were the second cause, while falls from height (n=2, 1.6%) and gunshot wounds (n=2, 1.6%) were the third cause among the cases. Sprain, direct trauma, and work accidents were registered for one patient each (0.8%). Trauma mechanism data were unavailable for one patient (0.8%).

**Table 2:** Fracture characteristics, trauma mechanisms, associated injuries, and management of fracture n = absolute frequencies; % = relative frequencies

Laterality	n (%)
Right	68 (54.4%)
Left	57 (45.6%)
Müller-AO classification	n (%)
42A	86 (68.8%)
42B	35 (28%)
42C	4 (3.2%)
Fracture type	n (%)
Open	24 (19.2%)
Closed	101 (80.8%)
Prior external fixation	n (%)
Yes	23 (18.4%)
No	102 (81.6%)
Associated fractures	n (%)
Yes	19 (15.2%)
No	106 (84.8%)
Traumas in other systems	n (%)
None	105 (84%)
Thoracic	2 (1.6%)
Abdominal	1 (0.8%)
No information	17 (13.60%)
Trauma mechanisms	n (%)
Traffic accident	105 (84%)
Sports trauma	4 (3.2%)
Physical assault	4 (3.2%)
Fall from height	2 (1.6%)
Ground-level fall	4 (3.2%)
Direct trauma	1 (0.8%)
Gunshot wound	2 (1.6%)
Sprain	1 (0.8%)
Work accident	1 (0.8%)
No information	1 (0.8%)

Most fractures (n=123, 98.4%) consolidated within six months, while two cases (1.6%) failed to consolidate after one year (Table 3). Complications occurred in four cases (3.2%), with one isolated malunion (0.8%), one infection (0.8%), and two nonunion (pseudoarthrosis) with screw breakage (1.6%).

**Table 3:** Postoperative outcomes n = absolute frequencies; % = relative frequencies

Consolidation (within six months)	n (%)
Yes	123 (98.4%)
No	2 (1.6%)
Consolidation (within one year)	n (%)
Yes	123 (98.4%)
No	2 (1.6%)
Complications	n (%)
None	121 (96.8%)
Infection	1 (0.8%)
Malunion	1 (0.8%)
Nonunion and screw breakage	2 (1.6%)

No comorbidities were found to be associated with the complications. Infection occurred in a patient with an open fracture and prior fixation who reported substance use (tobacco and alcohol); despite the infection, the patient achieved consolidation within six months. Nonunion with screw breakage was observed in two patients with closed fractures and without a history of substance use: one had previous external fixation, while the other did not. The patient with infection and the two patients with screw breakage and nonunion were indicated for revision surgery. Malunion occurred in a patient with a closed fracture, without a history of substance use, and with external fixation.

Subgroup analyses

There was a significant association between previous external fixation and type of fracture (closed and open) (𝝌²(1) = 46.1, p Fischer < 0.001, Table 4). Most patients with closed fractures (n=94, 93.1%) did not require previous external fixation, while only seven (6.9%) had undergone external fixation. Among patients with open fractures, 16 (66.7%) had a previous external fixation, while eight patients (33.3%) did not require external fixation. There was no significant association between previous external fixation and consolidation within one year post-surgery (𝝌²(1) = 1.35, p Fischer = 0.33, Table 5). Among patients who underwent external fixation, 22 (95.7%) consolidated at six months. Among those who did not have a previous external fixation, 101 (99.0%) of the fractures consolidated at six months. Only two patients did not consolidate, one with previous external fixation and the other without. Both patients did not consolidate after one year and presented pseudarthrosis and screw breakage as complications.

**Table 4:** Contingency table of previous fixation and type of fracture (closed and open) 𝝌²(1) = 46.1, p Fischer < 0.001, power (1-β err prob) = 0.99, actual α = 0.035

		Previous fixation		
Type of fracture		No	Yes	Total
Close	Observed	94	7	101
	% line	93.1 %	6.9 %	100.0 %
Open	Observed	8	16	24
	% line	33.3 %	66.7 %	100.0 %
Total	Observed	102	23	125
	% line	81.6 %	8.4 %	100.0 %

**Table 5:** Contingency table of previous external fixation and consolidation within one year 𝝌²(1) = 1.35, p Fischer = 0.33, power (1-β err prob) = 0.99, actual α =0.00

		Consolidation within one year		
Previous external fixation		Yes	No	Total
No	Observed	101	1	102
	% line	99.0 %	1.0 %	100.0 %
Yes	Observed	22	1	23
	% line	95.7 %	4.3 %	100.0 %
Total	Observed	123	2	125
	% line	98.4 %	1.6 %	100.0 %

## Discussion

This study evaluated the epidemiological profile of patients with tibial diaphyseal fractures and outcomes of IMN treatment. Our results demonstrated tibial diaphyseal fractures in a predominantly young male population. Outcomes are favorable, with low rates of complications. Additionally, we observed the association between open fractures and the previous use of external fixation.

The predominance of males in our sample (80%) is consistent with previous studies [2,12,15]. This is also in line with the majority of tibial fractures in male populations (87%) in low- and middle-income countries [4] and a male-to-female ratio of approximately 2:1 [3]. A bimodal age distribution with peaks at 20 and 50 years in tibial fractures [3] supports the conformity of our sample (mean age: 33.37 years) with global trends, as the mean age of patients in developing countries is typically under 30 years [14]. In contrast, in high-income countries, the average is closer to 40 years [18]. Studies involving Brazilian and Argentine populations reported a mean age of 36.2-37.5 years and a male predominance of 82.6-84.7% [17,19].

Demographic characteristics of the sample are also related to fracture trauma mechanisms. Mechanisms of trauma in tibial fractures include high-energy causes such as traffic accidents (motorcycles, cars, and pedestrian collisions), falls from height, and gunshot wounds, and low-energy causes such as ground-level falls and sprains [2,20]. Traffic accidents are the most frequent mechanism of trauma of diaphyseal fractures [21] and the first cause among younger populations [3], evidencing high-energy trauma in younger individuals. In contrast, low-energy trauma predominates in older populations [20]. Traffic accidents are the leading trauma mechanism in our sample (84%), similar to the 77.8% reported among Brazilian and Argentine populations [17]. The high incidence of these fractures in Brazil is related to the frequent use of motorcycles and may explain the demographic characteristics of the injured population [15,17]. High-energy trauma is correlated with fractures in other bones and high rates of injuries in different systems (49.5%) [15]. However, in our sample, only 15.2% of patients presented associated fractures, while 84% had no trauma involving other systems.

In high-energy tibial diaphyseal trauma, the incidence of nonunion is higher compared to low-energy trauma [22,23]. Two nonunions occurred in our sample six months post-surgery. Those two cases were the only fractures that failed to consolidate after one year. Open fractures, non-steroidal anti-inflammatory drugs, smoking, high-energy trauma, polytrauma, associated fibula fractures, interfragmentary gap over five mm, and superficial infection are risk factors for nonunion [24-27]. Although the two nonunions in our sample occurred in fractures caused by traffic accidents, the patients were neither smokers nor had associated fractures, the fractures were closed, and none had superficial infections. There is a strong association between delayed union and nonunion following previous external fixation [15,25]. However, we did not find this association in our results, and only one of the two nonunions in our cohort involved previous external fixation. Our data reveal an association between the previous use of external fixators and the occurrence of open fractures. External fixation is commonly used for open tibial fractures, mainly due to its ability to maintain bone stability [9,10]; therefore, this association is expected, given its relationship to the injury characteristics. A limitation of our study is the lack of a Gustilo-Anderson classification for open fractures, which limited our analysis of the association between complications and soft tissue injury.

Our study had a low complication rate. Malunion occurred in one case, and screw breakage and nonunion occurred in two patients. A single case of infection occurred in a patient with an open fracture and previous external fixation. Infections remain a significant complication in tibia fractures, identified as a key factor influencing return-to-work rates [16], and are related to fixation methods [28]. Soft-tissue reconstruction and previous external fixation are risk factors for infection in IMN of tibial and femoral fractures [16]. Those fracture characteristics are frequent in high-energy trauma. Trauma mechanisms and soft-tissue conditions are good predictors of complications in tibial diaphyseal fractures, with an increased risk of nonunion in high-energy trauma [23] and infections in severe soft-tissue injuries [24]. Thus, it is plausible that the association in the infection rates is more related to the trauma mechanism and injury characteristics rather than the use of external fixation. However, some fixation techniques, such as open reduction and internal fixation with osteosynthesis plates, present higher rates of infection than IMN [11].

Some of the complications of IMN can require another surgical intervention to guarantee bone healing. In a systematic review involving 6088 patients, 17.8% underwent subsequent surgeries, with rates ranging from 0% to 68% among the studies [29]. Screw removal due to pain or discomfort is the most common reason for reoperation (8.9%), while infection accounted for 1.2% of revision surgeries [29]. Nail revision (4.2%) and bone grafting (2.4%) are frequent indications of subsequent surgeries to promote bone union [29]. Independent predictors for revision surgeries were young age, multi-trauma, open fractures, weekday surgeries [19], and a lack of cortical continuity of more than 25% [30]. In our cohort, the patients with infection, nonunion, and screw breakage were indicated for revision surgery.

Although the outcomes of tibial diaphyseal IMN vary in recent literature, some of our findings are similar to those of previous reports. Regardless, as a retrospective multicenter study, our research has limitations. Different data on fracture characteristics, such as the severity of soft tissue injuries, were not considered and could have offered new perspectives for the analysis, indicating a need for future studies. Despite these limitations, our study shows the epidemiology and clinical outcomes of IMN from a multicentric perspective in two Brazilian cities over 3,000 kilometers apart, minimizing regional tendencies and addressing the existing literature, presenting new data to support and guide clinical decisions and public policies regarding the most critical victims of tibial fractures from a national perspective.

## Conclusions

Our sample was predominantly male and young, with traffic accidents being the leading cause of injury. In this context, the treatment outcomes in our study were favorable, with most patients achieving fracture consolidation within six months and low complication rates. IMN has proven to be an effective treatment, reaffirming its benefits in the Brazilian population, a country with an upper-middle-income economy. Some complications, such as infection, nonunion, and malunion, were seen; however, their incidence was low. These findings suggest that IMN is a safe and effective treatment for tibial diaphyseal fractures, with positive results in terms of fracture consolidation and low complication rates.
